# Prevalence of afebrile malaria and development of risk-scores for gradation of villages: A study from a hot-spot in Odisha

**DOI:** 10.1371/journal.pone.0221223

**Published:** 2019-09-06

**Authors:** Bhuputra Panda, Mrinal Kar Mohapatra, Saswati Paital, Sreya Kumbhakar, Ambarish Dutta, Shridhar Kadam, Subhash Salunke, M. M. Pradhan, Anil Khurana, Debadatta Nayak, R. K. Manchanda

**Affiliations:** 1 Indian Institute of Public Health, Bhubaneswar, Odisha, India; 2 Health and Family Welfare Department, Government of Odisha, Bhubaneswar, Odisha, India; 3 Central Council for Research in Homeopathy, Ministry of AYUSH, Government of India, New Delhi, India; Instituto Rene Rachou, BRAZIL

## Abstract

**Introduction:**

Malaria is a public health emergency in India and Odisha. The national malaria elimination programme aims to expedite early identification, treatment and follow-up of malaria cases in hot-spots through a robust health system, besides focusing on efficient vector control. This study, a result of mass screening conducted in a hot-spot in Odisha, aimed to assess prevalence, identify and estimate the risks and develop a management tool for malaria elimination.

**Methods:**

Through a cross-sectional study and using WHO recommended Rapid Diagnostic Test (RDT), 13221 individuals were screened. Information about age, gender, education and health practices were collected along with blood sample (5 μl) for malaria testing. Altitude, forestation, availability of a village health worker and distance from secondary health center were captured using panel technique. A multi-level poisson regression model was used to analyze association between risk factors and prevalence of malaria, and to estimate risk scores.

**Results:**

The prevalence of malaria was 5.8% and afebrile malaria accounted for 79 percent of all confirmed cases. Higher proportion of Pv infections were afebrile (81%). We found the prevalence to be 1.38 (1.1664–1.6457) times higher in villages where the Accredited Social Health Activist (ASHA) didn’t stay; the risk increased by 1.38 (1.0428–1.8272) and 1.92 (1.4428–2.5764) times in mid- and high-altitude tertiles. With regard to forest coverage, villages falling under mid- and highest-tertiles were 2.01 times (1.6194–2.5129) and 2.03 times (1.5477–2.6809), respectively, more likely affected by malaria. Similarly, villages of mid tertile and lowest tertile of education had 1.73 times (1.3392–2.2586) and 2.50 times (2.009–3.1244) higher prevalence of malaria.

**Conclusion:**

Presence of ASHA worker in villages, altitude, forestation, and education emerged as principal predictors of malaria infection in the study area. An easy-to-use risk-scoring system for ranking villages based on these risk factors could facilitate resource prioritization for malaria elimination.

## Introduction

Malaria, one of the highly prevalent infectious diseases, accounted for about 216 million new cases and 0.45 million deaths in 2016, globally [1]. The most common manifestation of malaria is the typical characteristic presentation a cyclical syndrome: fever, chill, sweat, headache, and vomiting—mostly in non-immune individuals. Yet another lesser known type of ‘asymptomatic’ malaria continue to exist amongst individuals who have had partial or complete immunity to the disease—mostly in population residing in malaria endemic areas [2,3]. Asymptomatic cases pose greater challenges to the program managers as they act as hidden reservoirs of active infection that perpetuates sustained transmission. In settings that adopt passive surveillance such as in India, these reservoirs pose formidable challenges for malaria elimination [4]. Therefore, strategies to eliminate asymptomatic infections would have greater public health consequences not only from the point of view of caseload but also achievement the elimination objectives.

India is a signatory to the National Framework for Malaria Elimination (NFME). Government of India in close alignment with the Global Technical Strategy has detailed a roadmap for implementation of the national strategy for malaria elimination as to achieve the national goals by 2030 [5]. Under this national framework, about 40.0% reduction of incidences and mortalities by 2020 is envisaged as compared to that of 2015. Interventions such as mass distribution of Long-Lasting Insecticidal Nets *(*LLINs), intensification of Indoor Residual Spray (IRS) and administration of Artemisinin-based Combination Therapy (ACT), have resulted in temporary reduction of malaria cases, mostly febrile, though such cases represent only the tip of the iceberg. Further, the national malaria elimination program adopts passive surveillance for case identification, treatment and reporting[6]. Thus, the chances of ‘asymptomatic cases’ going unnoticed continue to remain high. Identification of asymptomatic “hotspots” and the strategy of targeted interventions ought to have strong strategic, operational and policy implications.

Taiwan, China, and Brazil have resorted to active case detection and treatment (ACDT), also referred to as mass test and treatment (MTAT), in which mass screening of at-risk population is undertaken through rapid diagnostic test (RDT), followed by effective treatment of detected cases as to reduce transmission of Malaria [4]. However, owing to the nature of transmission, mass interventions for malaria control were historically short-lived [7] as they required incessant mass screening and treatment options to eliminate possibility of any outbreak. Therefore, malaria elimination needs a systematic and targeted ACDT approach, driven by context-specific evidences about ‘hotspots’. The Recent success in reduction of malaria in Odisha [8] need to be considered with a word of caution, as our earlier experiences in dealing with Malaria in the State has had mixed outcomes.

Therefore, this paper, the first of its kind, aims to present evidence on the prevalence of asymptomatic reservoir in one of the highly endemic and inaccessible pockets of Odisha [9], and to offer a ‘risk-score’ as an easy-to-use tool for program managers to rank villages as per existence of risk factors, which has the potential to facilitate malaria elimination in the State.

## Materials and methods

### Study design

A cross-sectional baseline mass screening for Malaria was conducted in 47 villages spread across four sub centres (SC) of Pallahara Block, Angul district, Odisha. The main objective was to estimate symptomatic and asymptomatic malaria cases in the community, using WHO recommended Rapid Diagnostic Test (RDT). We also aimed to identify the predictors that could explain the variation in the prevalence of malaria in these sample villages.

### Study setting and sampling

This study was conducted in four neighboring malaria endemic sub-centers of Pallahara block in Angul district of Odisha (21°29'N and 85°14'E). The average annual rainfall of Pallahara is reported 1551 mm. We used multistage stratified random sampling to sample the subcenters. First, 30 districts of the state were divided into four categories on the basis of reported API of 2016–17. Angul district was randomly selected from the top priority districts, reporting high API. In the second stage, three high API blocks were considered for Block selection. Pallahara Block was selected at random from the list of three. In the third stage, all the sub-centres in the selected block were divided into two groups based on their API and four sub-centres of Pallahara were chosen randomly from the higher API group. All villages of three sub-centres and seven villages, selected randomly, of the fourth sub-centre were included in the study. The villages had similar thick forest coverage and were dominated by tribal population. Data from the health department over past three years indicated P. Falciparum (Pf) as the predominant malarial species in that area. Mass screening was conducted in all 47 villages. A total of 13221 individuals out of 17552 enumerated population were screened.

### Study procedures

Eight field investigators (FI), 2 research assistants (RA) and one senior research assistant were hired and trained under the project. The field team was divided into four sub-teams of two FIs and one RA. The senior research assistant (SRA) cross checked the validity of data and confirmation of fever cases in community. Screening was done in camps and through door-to-door visit for the left-out population. Rapid diagnostic tests were performed using WHO-approved EzDx antigen Pf/Pv kit manufactured by ADVY chemicals, India at the field sites that contained a monoclonal anti-P. Falciparum histidine-rich protein II (HRP-II) specific antibody and an anti-P. Vivax (Pv) p-LDH-specific antibody to detect PF and PV malarial parasite infections. All positive cases were treated with an artemisinin-based combination therapy (ACT), a combination of artesunate (AS) plus sulfadoxine-pyrimethamine (SP), as per the National Vector Borne Disease Control Program (NVBDCP) guidelines.

### Definitions

A test using RDT is considered malaria positive if either or all of the two test bands (PF test line and PV test line) along with the control band are noticeable. A case of febrile malaria was defined as an individual with a history of fever within the past 48 hrs and with an axillary temperature of >37.5°C at the time of survey, while an afebrile case of malaria was defined as absence of fever within the past 48 hrs and an axillary temperature ~37.5°C at the time of survey.

### Data collection and management

A client-centric register was used for data collection. Questions related to basic demographic characteristics, such as, age, gender, education and health practices were asked. Blood samples (5 μl) were collected from the individuals to test PF and PV infection. Further, data related to village characteristics (eg., altitude, forestation, availability of a village health worker, distance from sub centre (SC) and primary health centre (PHC), etc) were collected in a separate register through expert-group consultations. The expert groups for each village consisted of representatives from the forest department, health department and senior citizens. Similarly, Altimeter Android application was used to measure the altitude of each village.

### Statistical analysis

Distribution of fever cases, education, mosquito net use and all the other relevant co-variates were examined in descriptive tables. Statistical significance of the distribution differentials was tested using chi-square test. Further, the individual data was aggregated to calculate the village wise malaria prevalence (which is our principle outcome variable) and average education.

To estimate the association, we modelled the principle outcome variable using a multilevel model to account for the clustered nature of the data, i.e. villages were clustered within a SC. We used the poisson regression framework in the screening population as offsetting variable for modelling the unadjusted, the adjusted and the final analysis. The final model was constructed based on statistical significance of variables in unadjusted and fully adjusted models. Through fully-adjusted estimations, the impact of individual explanatory variables on the estimates of regression were measured using marginal effect of each variable at a given level, keeping other variables at average—also called Average Marginal Effect (AME)—this is an intuitive method to interpret complex outcomes of a general linear model with log links.

The marginal effect measures the change in the expected value of y as one independent variable increases by unity while all other variables are kept constant. Therefore, the average marginal effect computes the average of all the individual marginal effects[10]. We computed the final poisson model and then obtained the average marginal effects for all covariates by using the command “margins” available in package “margins” for R-software. The formula to compute AMEs for the *i*th explanatory variable is

$$\frac{1}{n}\sum_{k=1}^{n}\{F(\beta x^{k}{+\beta }_{i})-F(\beta x^{k})\}$$

Where *βx^k^* denotes the linear combination of parameters and variables for the *k* th observation.

        *F*(.) denotes the cumulative distribution function.

We created algorithms based on the AMEs in order to rank and score the villages according to their probability of reporting higher prevalence. The scores against each factor were then summed up to create a village score which was used to rank the villages—the highest ranked village being the most risk-prone.

### Ethical issues

The study obtained ethics approval from the Institutional Review Board of Indian Institute of Public Health, Bhubaneswar and subsequently from the State Research and Ethics Committee of Government of Odisha. Written informed consent and assent (translated into the local language) was obtained from all participants. No material benefits were offered to any of the study participants. Confirmed malaria cases were treated on the spot by suitable anti-malarial drugs and referred to the government institutions. Identities of all participants were anonymized using identity numbers and the decode keys were maintained only by the principal investigator.

## Results

Our principal explanatory variables were village characteristics that posed a greater risk for malaria transmission and infection: availability of a village health worker (Accredited Social Health Activist), altitude of the village, percentage of land covered by forestation, distance of village from the nearest SC and the PHC, use of long-lasting Insecticidal nets (LLIN) and average years of education (Table 1).

**Table 1:** Principal explanatory variables.

Variable	Value	Malaria prevalence n (%)
**Asha**		
Staying in the village	21 (44.7)	379(0.49)
Not staying in the village	26 (55.3)	394(0.50)
**Altitude in meter**		
Median (IQR)	186 (168.5–205)	
**Tertile range**		
Lowest tertile	147–174.9	92(0.11)
Mid tertile	175–196.9	300(0.38)
Highest tertile	197–287	381(0.49)
**Forestation percentage**		
Median (IQR)	40 (22.50–62.50)	
**Tertile range**		
Lowest tertile	0–29.9	136(0.17)
Mid tertile	30–59.9	327(0.42)
Highest tertile	60–90	310(0.40)
**Distance from secondary healthcare facility**		
Median (IQR)	15.50 (12.00–18.50)	
**Tertile range**		
Lowest tertile	7.0–13.9	252(0.32)
Mid tertile	14–17.69	199(0.25)
Highest tertile	17.7–29	322(0.41)
**Average years of education of village**		
Median (IQR)	3.3 (2.52–4.28)	
**Tertile range**		
Highest tertile	4.2–5.86	171(0.22)
Mid tertile	2.58–4.19	265(0.34)
Lowest tertile	1.3–2.579	337(0.43)
**Proportion of respondents using LLIN regularly**		
Median (IQR)	75.94 (67.57–79.94)	
**Tertile range**		
Highest tertile	1–0.951	234(0.30)
Mid tertile	0.951–0.906	306(0.39)
Lowest tertile	0.906–0	233(0.30)

We found hardly any difference in gender distribution across groups, while age distribution was significantly different. P. falciparum prevalence was more among 5–14 years age group, while Pv was prevalent among under-five children. The mean years of education was significantly higher among non-infected (3.7 years) than Pf infected (2.6 years), Pv infected (2 years) and mixed infected (2.4 years) population. Further, infection was significantly lower among professionals, skilled labors and homemakers and was comparatively higher among students and unemployed. Afebrile fever cases were more among Pv infected (81%) individuals than with Pf and mixed-infection individuals (Table 2).

**Table 2:** Individual demographic characteristics.

	Negative	Plasmodium Falciparum	Plasmodium Vivax	Mixed Infection	Total	P-value
	No. 12,448	No. 672	No. 60	No. 41	No. 13,221	
**Gender**						
Male	5,931 (47.6%)	333 (49.6%)	21 (35.0%)	19 (46.3%)	6,304 (47.7%)	0.18
Female	6,517 (52.4%)	339 (50.4%)	39 (65.0%)	22 (53.7%)	6,917 (52.3%)	
**Age**						
0 to 4 yrs	1,372 (11.0%)	110 (16.4%)	17 (28.3%)	8 (19.5%)	1,507 (11.4%)	< 0.0001
5 to 14 yrs	2,050 (16.5%)	186 (27.7%)	13 (21.7%)	8 (19.5%)	2,257 (17.1%)	
15 to 59 yrs	8,149 (65.5%)	340 (50.6%)	29 (48.3%)	24 (58.5%)	8,542 (64.6%)	
60 and above	877 (7.0%)	36 (5.4%)	1 (1.7%)	1 (2.4%)	915 (6.9%)	
**Marital status**						
Unmarried	5,153 (41.4%)	366 (54.5%)	36 (60.0%)	20 (48.8%)	5,575 (42.2%)	< 0.0001
Married	6,777 (54.4%)	285 (42.4%)	23 (38.3%)	20 (48.8%)	7,105 (53.7%)	
Separated	11 (0.1%)	1 (0.1%)	0 (0.0%)	0 (0.0%)	12 (0.1%)	
Divorced	11 (0.1%)	1 (0.1%)	0 (0.0%)	0 (0.0%)	12 (0.1%)	
Widowed	496 (4.0%)	19 (2.8%)	1 (1.7%)	1 (2.4%)	517 (3.9%)	
**Education categories**						
Median (IQR)	2.0 (0.0–7.0)	0.0 (0.0–5.0)	0.0 (0.0–2.0) 2.0 (±3.6)	0.0 (0.0–3.0)	2.0 (0.0–7.0)	< 0.0001
Illiterate	5,542 (44.5%)	349 (51.9%)	35 (58.3%)	22 (53.7%)	5,948 (45.0%)	< 0.0001
Primary	3,005 (24.1%)	189 (28.1%)	17 (28.3%)	13 (31.7%)	3,224 (24.4%)	
Secondary	3,204 (25.7%)	118 (17.6%)	5 (8.3%)	4 (9.8%)	3,331 (25.2%)	
Intermediate	439 (3.5%)	9 (1.3%)	2 (3.3%)	1 (2.4%)	451 (3.4%)	
Graduation and Above	258 (2.1%)	7 (1.0%)	1 (1.7%)	1 (2.4%)	267 (2.0%)	
**Occupation categories**						
Professional	32 (0.3%)	0 (0.0%)	0 (0.0%)	0 (0.0%)	32 (0.2%)	< 0.0001
Service holder	345 (2.8%)	6 (0.9%)	0 (0.0%)	1 (2.4%)	352 (2.7%)	
Own business/farming	2,619 (21.0%)	131 (19.5%)	11 (18.3%)	11 (26.8%)	2,772 (21.0%)	
Skilled labor	232 (1.9%)	7 (1.0%)	0 (0.0%)	0 (0.0%)	239 (1.8%)	
Casual labor	1,847 (14.8%)	85 (12.6%)	5 (8.3%)	6 (14.6%)	1,943 (14.7%)	
Agricultural labor	632 (5.1%)	32 (4.8%)	6 (10.0%)	2 (4.9%)	672 (5.1%)	
Home maker	2,043 (16.4%)	71 (10.6%)	3 (5.0%)	4 (9.8%)	2,121 (16.0%)	
Student	2,295 (18.4%)	167 (24.9%)	16 (26.7%)	7 (17.1%)	2,485 (18.8%)	
Unemployed	2,403 (19.3%)	173 (25.7%)	19 (31.7%)	10 (24.4%)	2,605 (19.7%)	
**Mosquito net use**						
Always	11,378 (91.4%)	568 (84.5%)	52 (86.7%)	38 (92.7%)	12,036 (91.0%)	< 0.0001
Irregularly	721 (5.8%)	75 (11.2%)	7 (11.7%)	2 (4.9%)	805 (6.1%)	
Never	349 (2.8%)	29 (4.3%)	1 (1.7%)	1 (2.4%)	380 (2.9%)	
**Fever status during screening**						
Febrile	874 (7.0%)	142 (21.1%)	11 (18.3%)	10 (24.4%)	1,037 (7.8%)	< 0.0001
Afebrile	11,574 (93.0%)	530 (78.9%)	49 (81.7%)	31 (75.6%)	12,184 (92.2%)	

**Fig 1:**
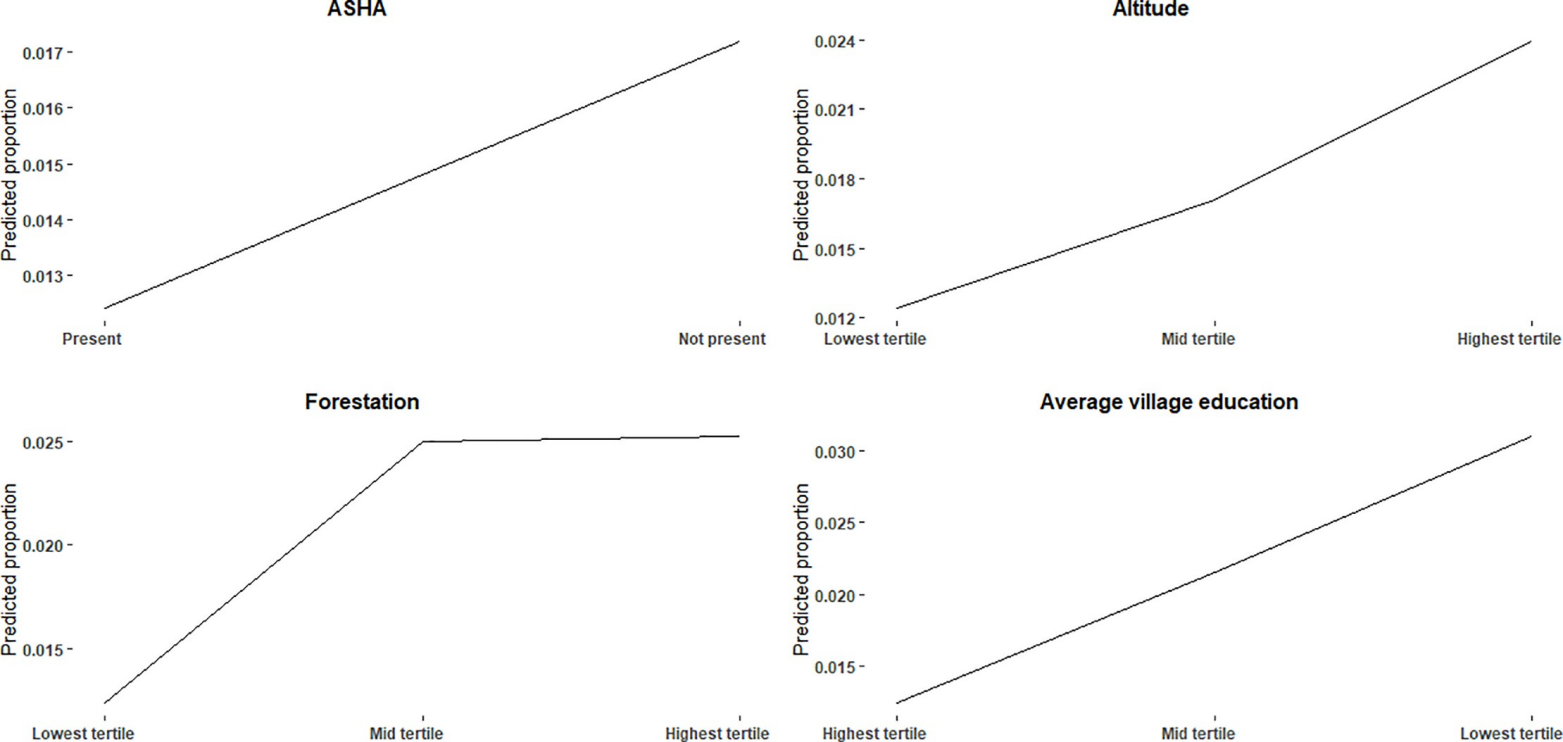
Predicted proportion for Malaria.

Afebrile malaria accounted for 79 percent of all confirmed cases. And the prevalence of asymptomatic malaria among adults is higher than that of 0–5 years group. Four out of six independent variables were found to be significantly associated with prevalence of malaria in the final model. We found the prevalence 1.38 times higher in villages where ASHA didn’t stay as compared to villages where ASHA resided. Further, the risk increased by 1.38 and 1.92 times in mid- and high-altitude tertiles. With regard to forest coverage, villages falling under mid- and highest-tertiles were 2.01 times and 2.03 times, respectively, more likely to have been affected by malaria than villages under the lowest tertile. Similarly, villages of mid tertile and lowest tertile of education had 1.73 times and 2.50 times higher prevalence of malaria than those in the highest tertile (Table 3). These findings are also reflected in Fig 1.

**Table 3:** Association of village characteristics with prevalence of malaria.

	Prevalence ratio		
	Unadjusted Risk Ratio	Fully Adjusted Risk ratio	Final model
**ASHA**	*	*	*
Staying in the village	1	1	1
Not staying in the village	2.2284 (1.9203, 2.5858)	1.3748 (1.1566, 1.6341)	1.3855 (1.1664, 1.6457)
**Altitude of village**	*	*	*
Lowest tertile	1	1	1
Mid tertile	1.6918 (1.3263, 2.1582)	1.3372 (1.0016, 1.7851)	1.3803 (1.0428, 1.8272)
Highest tertile	3.5145 (2.7452, 4.4994)	1.8557 (1.3631, 2.5263)	1.928 (1.4428, 2.5764)
**Forest coverage in village**	*	*	*
Lowest tertile	1	1	1
Mid tertile	1.8413 (1.4846, 2.2836)	2.0212 (1.6204, 2.5211)	2.0173 (1.6194, 2.5129)
Highest tertile	3.4169 (2.6999, 4.3243)	2.0132 (1.5300, 2.6491)	2.0369 (1.5477, 2.6809)
**Distance from SDH (Kms)**	*	#	
Lowest tertile	1	1	-
Mid tertile	1.2764 (1.0546, 1.5448)	0.9423 (0.7706, 1.1522)	-
Highest tertile	2.0466 (1.7326, 2.4174)	1.1624 (0.9558, 1.4135)	-
**Average education**	*	*	*
Highest tertile	1	1	1
Mid tertile	2.5859 (2.1154, 3.1609)	1.7006 (1.3094, 2.2086)	1.7392 (1.3392, 2.2586)
Lowest tertile	3.4244 (2.829, 4.145)	2.3916 (1.9048, 3.0029)	2.5054 (2.009, 3.1244)
**Mosquito net useas**	#		
Highest tertile	1	-	-
Mid tertile	1.5594 (1.3058, 1.8623)	-	-
Lowest tertile	1.0880 (0.8971, 1.3194)	-	-

* Statistically significant at an alpha level of 0.05

# Statistically insignificant at an alpha level of 0.05

The marginal effect from fully adjusted model showed that availability of ASHA in villages played a critical role in the prevalence of malaria. Non-residence of ASHA at villages contributed to 2.2 percent increase in malaria prevalence across the study sites. Similarly, mid- and highest-altitude tertiles had 1.017 and 1.042 AME as compared to the lowest tertile. The AME of medium forested villages and highly forested villages on Malaria prevalence was approximately 1.041. Finally, the AME for mid- and lowest-education tertiles were 1.028 and 1.056, respectively (Table 4).

**Table 4:** Average marginal effects.

Factor	AME	AME (%)	SE	z	p	lower	upper
**ASHA**							
Staying in the village	**0**	0					
Not staying in the village	0.022	2.22	0.006	3.4724	< 0.001	0.0097	0.0347
**Altitude**							
Lowest tertile	0	0					
Mid tertile	0.017	1.73	0.008	2.2843	0.022	0.0025	0.0321
Highest tertile	0.042	4.21	0.01	4.2953	< 0.001	0.0229	0.0614
**Forested area**							
Lowest tertile	0	0					
Mid tertile	0.04	4.02	0.008	5.183	< 0.001	0.025	0.0554
Highest tertile	0.041	4.1	0.01	4.2489	< 0.001	0.0221	0.0599
**Average education of villagers**							
Highest tertile	0	0					
Mid tertile	0.027	2.76	0.007	3.8315	0.001	0.0135	0.0417
Lowest tertile	0.056	5.62	0.01	5.8396	< 0.001	0.0373	0.075

### Risk score estimation

We used poisson regression with population as offset to model the association of independent variables of interest. Though this model offers flexibility to specify potentially complex multivariate relationships, it also brings the risk of misinterpretation and miscalculation [11,12]. Moreover, the coefficient estimates derived from such generalized linear models (GLM) don’t provide unconditional marginal effects and therefore lose their direct interpretational value. Hence, in order to make the interpretations more intuitive, we used ‘average marginal effects’ estimate as it provides information about the rate at which the dependent variable changes at a given point in the covariate space with respect to one covariate dimension while holding all other covariate values constant [13,14]. To rank villages according to their probability of reporting high prevalence of malaria, we multiplied the AMEs with 100 to get the effect on a percentage scale which were rounded off to make the ranking tool easier for use (Table 5).

**Table 5:** Village risk score.

factor	Score
**ASHA**	
Staying in the village	0
Not staying in the village	2
**Altitude**	
Lowest tertile (147mts– 175mts)	0
Mid tertile (175mts– 197mts)	1.5
Highest tertile (197mts– 287mts)	4
**Forested area**	
Lowest tertile (0% - 30%)	0
Mid tertile (30% - 60%)	4
Highest tertile (60% - 90%)	4
**Average education of villagers**	
Highest tertile (4.2 yrs—5.86 yrs)	0
Mid tertile (2.58yrs—4.2yrs)	2.5
Lowest tertile (1.3yrs—2.58yrs)	5.5

The risk estimation table (Table 5) gives specific scores to a village according to its place in the spectrum of risk factors. For example, a village in third tertile of altitude, second tertile of forestation and second tertile of average education with no ASHA residing in it will get scores of: 4, 4, 2.5 and 2, respectively, and a cumulative score of 12.5. All villages in a given geographic area could be scored and ranked from the highest to the lowest rank and prioritized from the point of anti-malaria programme implementation.

## Discussion

This paper examined the prevalence of malaria, especially afebrile cases in one of the hotspots (Pallahara block) of Odisha, the state contributing maximum caseload and mortalities in India. It also analyzed the village level risk factors that influenced malaria prevalence. The results highlight a disproportionately high prevalence of afebrile cases. Further, availability of ASHA at village level had a direct and significant association with prevalence of malaria–this held true even after adjusting for known covariates such as altitude, forestation and education each of which had significant associations as well. The prevalence of afebrile malaria in all four SC areas was alarming, though the scenario may not be similar across all hotspots in Odisha.

A study conducted in the Kondagaon district of Chhattisgarh in India, a very similar terrain, found 65% asymptomatic cases among all positive cases [15]. Similarly another study from west Bengal found a very high prevalence of asymptomatic malaria (8.4%) in healthy tribal population in a malaria endemic area [16]. The burden of such asymptomatic malaria in endemic areas can be attributed to high herd immunity and low density of the infection [17]. Further, such findings are “textbook” examples of the “Iceberg” phenomena [18] and suggest that the clinically ill cases are only a handful of what lies beneath. No malaria elimination campaign would achieve the objectives without addressing these sub-clinical infections. This further emphasizes the importance of ACDT to detect and stop transmission of malaria infection.

Higher prevalence of infection was observed among children (0–14 yrs) as compared to adults that implies development of protective immunity with age. Several past studies re-affirm that acquired immunity increases with age, leading to higher prevalence of asymptomatic cases among adults [2,15,16,19,20]. Education as a principal factor for determining health outcomes was also found to be strongly associated with malaria prevalence [21–24].

Residence of ASHA, altitude, forestation and average education of village were found to be critical factors contributing to prevalence of malaria. Past studies have demonstrated the impact of community level health workers on prevalence of Tuberculosis (TB) and childhood mortality [25–28]. Ours is the first study showing strong association of ASHA’s residence with prevalence of malaria. It is also logical to relate the role of ASHA (screening fever cases, treating and monitoring) with anti-malaria programme implementation in a village. If an ASHA worker doesn’t stay in a village, these functions are likely to be delayed or disturbed.

Studies on malaria and altitude dynamics reveal that a fall in temperature is not conducive for vectors that transmit malaria [29,30], but in our study there is a direct association between altitude and prevalence of malaria–this may be mainly due to the fact that the range of altitude variation in this study settings is quite narrow, rather acts more as a proxy to forest dwelling for food habits, lesser acceptance of preventive measures leading to uninterrupted malaria transmission [31–34].

It is well established that forest ecosystem promotes vector sustainability and malaria transmission. A global assessment by Guerra et.al estimated that half of the risks for malaria is attributable to people living in forested areas [35]. Factors such as rainfall [36,37], humidity [38–40], tree canopy [41], and high organic content in breeding pools [31] in these forest regions influence malaria transmission as compared to other regions.

Finally, number of years of education emerged as one of the strongest associated factors, among all, with a clear gradient across tertiles. Education is a crucial social determinant of health across the globe [42] and it’s no different for Odisha. The strength of association between malaria and education need to be seen from the perspective of delivering better education to residents of hotspots which could potentially influence the probability of a sustained anti-malaria campaign and achievement of malaria elimination goals of the State. With education the health seeking behavior among the general public in such areas would also improve significantly [43], where educational attainment is poor in general.

Identification of risk factors and strengths of associations offered a unique opportunity to device an easy-to-use scoring tool for managers and administrators to rank villages which would have a huge implication in utilizing the limited resources efficiently in similar settings. Past studies have highlighted about the importance of predicting and placing early warning systems to combat malaria [36] as well as to delineate hotspots [44]. Our tool has the potential to add a chapter to further prioritization of high burden villages in hotspots for evolving a sustained and effective malaria elimination strategy.

### Limitations

This is the first ever study in one of the hotspots of Odisha in terms of mass screening of the entire population living under the sample clusters. The tool developed for risk-scoring of villages is also a unique product. However, it has some limitations as well: the study was conducted in a typical tropical region with moderately elevated lands: generalization of findings to a wider population, especially hilly terrains should be done with caution. Secondly, we have used RDTs that have limited detectability of low parasitemia, and hence, the prevalence of asymptomatic malaria as found in this study might be lower than if tested with Polymerase Chain Reaction (PCR)-based kit. Finally, temperature variations and existence of water bodies have not been included in this analysis, which have effects on vector breeding and malaria transmission.

## Conclusion

Presence of ASHA worker in villages, altitude, forestation, and education are strongly associated with malaria prevalence in a village. An easy-to-use risk-scoring system for ranking villages and prioritizing resource allocation for malaria elimination has the potential to change the paradigm of government’s approach in addressing malaria in the State of Odisha. Further in-depth investigation into the socio-cultural and behavioral patterns of people living in hotspots could through more light into the phenomenon of vector breeding and malaria transmission in the State.
